# Effect of human secretory calcium-binding phosphoprotein proline-glutamine rich 1 protein on *Porphyromonasgingivalis* and identification of its active portions

**DOI:** 10.1038/s41598-021-02661-w

**Published:** 2021-12-09

**Authors:** Charline Mary, Aurélien Fouillen, Pierre Moffatt, Dainelys Guadarrama Bello, Rima M. Wazen, Daniel Grenier, Antonio Nanci

**Affiliations:** 1 Laboratory for the Study of Calcified Tissues and Biomaterials, Faculty of Dental Medicine, Université de Montréal, Montréal, Québec H3T 1J4 Canada; 2 Department of Biochemistry and Molecular Medicine, Faculty of Medicine, Université de Montréal, Montréal, Québec H3T 1J4 Canada; 3 Department of Human Genetics, McGill University, Montreal, Québec H3A 0G4 Canada; 4 Shriners Hospitals for Children—Canada, Montreal, Québec H4A 0A9 Canada; 5 Oral Ecology Research Group, Faculty of Dental Medicine, Université Laval, Québec, Québec G1V 0A6 Canada

**Keywords:** Peptides, Antimicrobials, Electron microscopy, Biological fluorescence

## Abstract

The mouth environment comprises the second most significant microbiome in the body, and its equilibrium is critical in oral health. Secretory calcium-binding phosphoprotein proline-glutamine rich 1 (SCPPPQ1), a protein normally produced by the gingival epithelium to mediate its attachment to teeth, was suggested to be bactericidal. Our aim was to further explore the antibacterial potential of human SCPPPQ1 by characterizing its mode of action and identifying its active portions. In silico analysis showed that it has molecular parallels with antimicrobial peptides. Incubation of *Porphyromonas* *gingivalis*, a major periodontopathogen, with the full-length protein resulted in decrease in bacterial number, formation of aggregates and membrane disruptions. Analysis of SCPPPQ1-derived peptides indicated that these effects are sustained by specific regions of the molecule. Altogether, these data suggest that human SCPPPQ1 exhibits antibacterial capacity and provide new insight into its mechanism of action.

## Introduction

The oral microbiome is the second most important in humans after that of the gut^1^. It comprises about 700 varieties of microorganisms, including bacteria, fungi, viruses and protozoa, with bacteria constituting the main inhabitants^2^. This complex microbiome is important in maintaining oral health; loss of its delicate equilibrium due to the propagation of specific periodontal pathogens can lead to periodontal diseases (PD), an inflammatory condition affecting the tooth supporting tissues. Mild and moderate forms of PD affect about 80% of the adult population worldwide and severe ones up to 15%^2–4^. In addition to oral manifestations, PD are linked to systemic complications that are life-threatening including diabetes, pulmonary disorders^3^ and colorectal cancers^5^. There is also evidence of a link to Alzheimer’s disease^2–4,6^. Oral bacteria can even translocate and colonize the gut to influence its microbiome and cause dysbiosis^7,8^. The above considerations support the capital importance of the oral environment for human health.

The junctional epithelium (JE) is the specialized portion of the gingiva that seals off the tooth supporting tissues from the aggressive oral environment via a specialized basal lamina (sBL) that has adhesive properties^9^. It was demonstrated that *Porphyromonas* *gingivalis* (*P.* *gingivalis*), a major periodontal pathogenic bacteria, degrades the components of the sBL except for one called secretory calcium-binding phosphoprotein proline-glutamine rich 1 (SCPPPQ1) protein^10^. In addition to its apparent resistance against bacterial degradation, rat SCPPPQ1 protein was unexpectedly shown to affect the integrity of the cell membrane of *P.* *gingivalis*^11^. In this study, we first carried out detailed informatic analysis on the human protein and used it to further explore the antibacterial potential of human SCPPPQ1 and define its mechanism of action using complementary imaging methods. Small peptides derived from the full-length human SCPPPQ1 protein were also created to identify the portions responsible for its antibacterial capacity. The study was also extended to other periodontal-relevant bacterial species. Our data demonstrates that human SCPPPQ1 exhibits parallels with antimicrobial peptides (AMPs) and is able to decrease the bacterial populations by aggregating bacteria and disrupting their membrane.

## Results

### Characterization of the human SCPPPQ1 amino acid sequence

**Figure 1:**
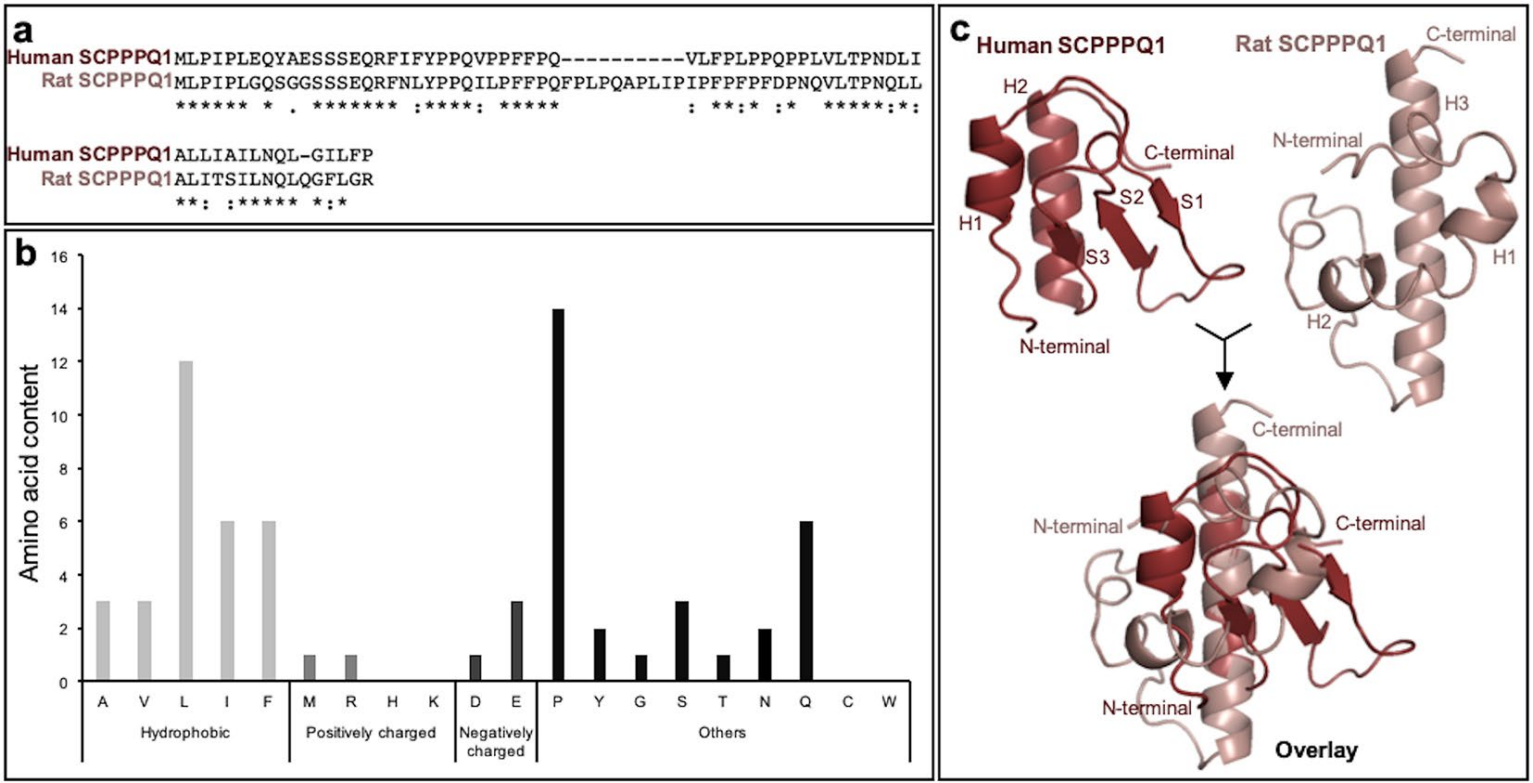
Characterization of the human SCPPPQ1 amino acid sequence. (**a**) Comparison of synthetic human and rat SCPPPQ1 amino acid sequences using MUSCLE. Sequence similarity is indicated as follow: (*) identical residue (single, fully conserved), (:) conserved substitutions (strong similar properties), and (.) semi-conserved substitutions (weak similar properties). (**b**) In silico analysis of the human SCPPPQ1 protein using the APD3 software provided information on the distribution and relative percentage of amino acids with hydrophobic properties, positive and negative charge, and other amino acids. (**c**) Tridimensional structure model from i-TASSER structure prediction software for human (red) and rat (pink) SCPPPQ1 proteins and their overlay. Secondary structure elements are included (H Helix, S Sheet) and labeled according to their position in the structure (H1 to H3, S1 to S3).

The human SCPPPQ1 amino acid sequence was recently reported by Moffatt and Nanci (GenBank MK322956.1; https://www.ncbi.nlm.nih.gov/nuccore/MK322956.1). Because a previous report on the antibacterial effect of SCPPPQ1 was based on the rat protein^11^, we used it as a reference for comparison with the human protein. CLUSTAL multiple sequence alignement tool MUSCLE (3.8) was applied to determine sequence similarity between the two mature proteins (Fig. 1a). The human protein is missing the amino acid sequence 32–41 that corresponds to exon 5 in rat, and the two proteins overall have 56% identity and 64% similarity (Fig. 1a). Analysis of the amino acid content indicated that the human SCPPPQ1 is rich in proline (22%), glutamine (9%), as well as hydrophobic residues, especially leucine (19%) (Fig. 1b). The overall hydrophobic amino acid content (42%) contributes to the grand average hydropathy value (GRAVY) of 0.45. The human SCPPPQ1 protein has a negative charge of -3 compared to the rat protein that has a neutral charge at pH 7. Structural predictions using i-TASSER and Quark revealed significant differences including (*a*) the presence of two α-helices (H1 and H2) and three ß-sheets (S1, S2 and S3) for the human protein, while the rat protein comprises three α-helices (H1, H2 and H3) and no ß-sheets and (*b*) the length of the C-terminal α-helix H2 of the human protein is shorter than the C-terminal α-helix H3 from the rat (Fig. 1c). GlobPlot 2.3 indicated that, in both cases, the predicted structures are highly disordered. These results indicate that the rat and human SCPPPQ1 proteins show major molecular differences and, therefore, results from the rat protein^11^ cannot be directly extrapolated to the human protein. However, like the rat protein, human SCPPPQ1 was predicted to possess potential anti-microbial peptide sequences as revealed by three different prediction algorithms that are available online (iAmpPred, AmPEP and Deep-AmPEP30)^12,13^. Analysis with the AMP database APD3 (University of Nebraska, Omaha, NE, USA)^14^ gave similar results.

### Effect of human SCPPPQ1 on *P. gingivalis*

**Figure 2:**
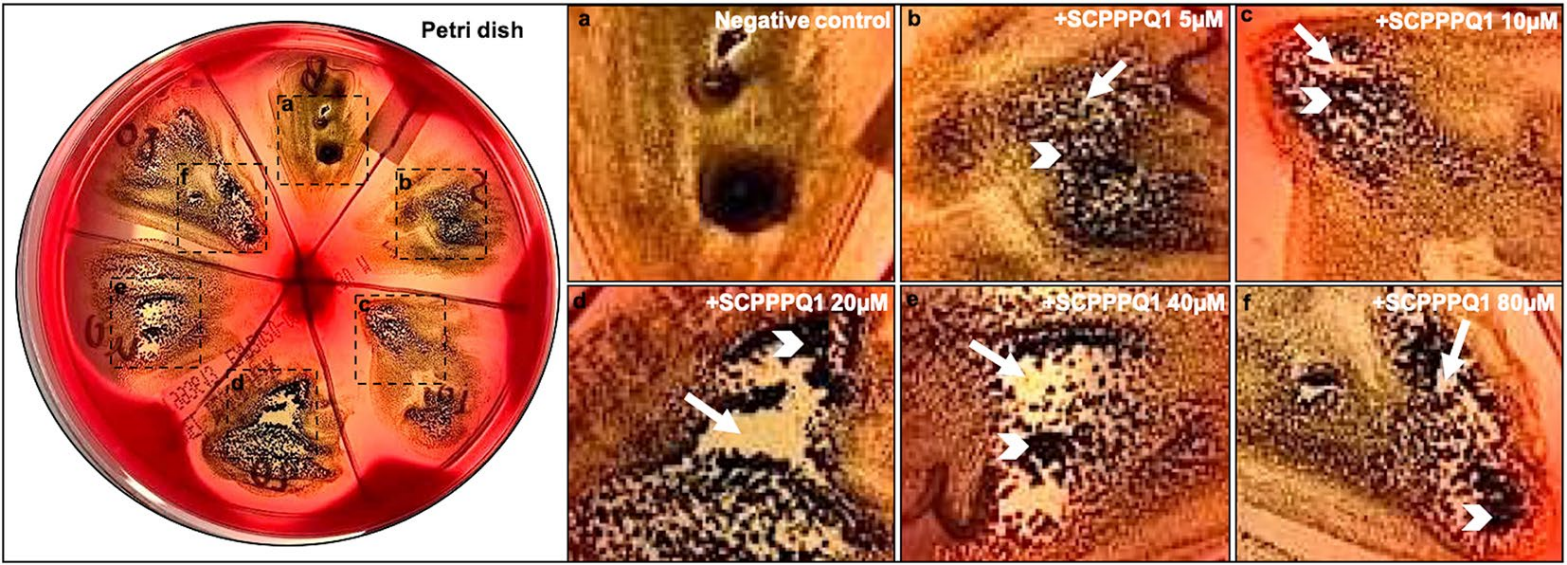
Determination of the working concentration of SCPPPQ1 that affect *P.* *gingivalis* growing conditions. Comparison of the difference in growth of the bacteria on a blood agar petri dish in (**a**) the presence of buffer alone (negative control) or (**b**–**f**) various concentrations of + SCPPPQ1. (**b**–**f**) Enlargements of the boxed areas in the blood agar plate (left panel). (**a**) Under control conditions the bacteria formed a relatively uniform layer that appeared black in photographs, (**b**–**f**) while in presence of proteins, this layer was interrupted producing a mosaic of black areas containing bacteria (white arrowhead) and white areas of various sizes that indicate absence/paucity of bacteria (white arrow). Qualitative evaluation suggests that the effect of SCPPPQ1 on the bacteria (white areas) started at 5 µM (weak) and plateaued at 20 µM (see Table 1). These results are representative of three experiments.

To test the antibacterial propensity of the synthetic human SCPPPQ1 on *P.* *gingivalis,* we have attempted (i) to determine the minimum inhibitory concentration (MIC) using a broth microdilution assay, and (ii) to perform a killing assay of planktonic cells. However, given that the SCPPPQ1 has to be solubilized in a urea buffer, these assays did not allow any conclusions since the buffer itself was found to be bactericidal in these specific conditions (see Supplementary materials). As an alternative method, a qualitative bactericidal assay on a blood agar plate was performed. In this assay, *P.* *gingivalis* was mixed with SCPPPQ1 and then applied on the surface of the agar plate. In the negative control (buffer without the protein), growth of the bacteria resulted in a uniform layer that appeared black on the blood agar plates, while areas without bacteria appeared white. As seen in the representative assay (Fig. 2), the extent of disruption of the uniform layer was more important when the SCPPPQ1 concentration was increased. As reported in Table 1, *P.* *gingivalis* was clearly affected at concentrations of 20 μM and above, although some sensitivity was also apparent with concentrations as low as 5 μM.

**Table 1:** MIC values of SCPPPQ1 against *P.* *gingivalis.*

SCPPPQ1 concentration (µM)	0	5	10	20	40	80
*P.* *gingivalis* sensitivity	–−	+−	+	+++	+++	+++

–− corresponds to a SCPPPQ1 concentration resulting in no effects on *P.* *gingivalis* growth while + + + is a concentration showing no growth of the microorganism.

**Figure 3:**
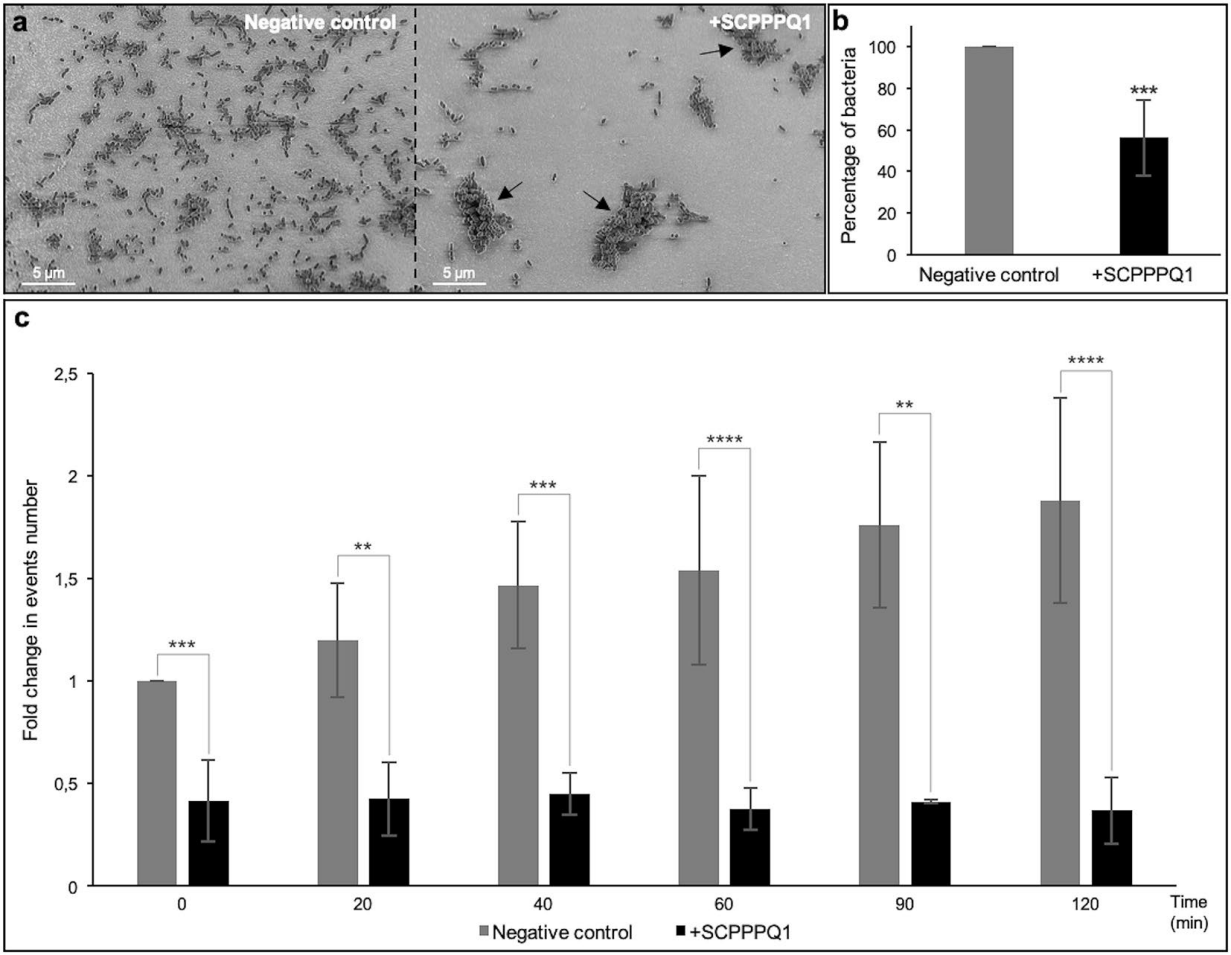
Effect of SCPPPQ1 on *P.* *gingivalis* bacterial population size. (**a**) SEM images showing bacteria from the negative control and + SCPPPQ1 treated sample at 20 min. Note the presence of aggregates only in presence of the protein (arrows). Micrographs are representative from five different experiments. (**b**) Quantification of the number of bacteria in percentage observed in SEM images incubated with + SCPPPQ1 normalized on the negative control at 20 min. Data are represented as mean ± standard error of mean (*n* = 5). Significance was determined by two-tailed Student’s *t* test analysis (***p < 0.001). (**c**) FACS results of fold change in events number over time, in samples treated with negative control or + SCPPPQ1, normalized on the negative control at 0 min. Data are represented as mean ± standard error of mean (*n* = 4). Significance was determined by two-tailed Student’s *t* test analysis (**p < 0.01; ***p < 0.001; ****p < 0.0001).

To characterize in greater detail the antibacterial propensity of the synthetic human SCPPPQ1 on *P.* *gingivalis,* we analysed the effect of the protein on the number of bacteria. Qualitative (Fig. 3a) and quantitative analysis of scanning electron microscope (SEM) images (Fig. 3b) revealed a significant decrease of 44% in the bacterial number after 20 min of incubation with respect to the buffer without the protein (negative control). The images also showed the presence of aggregates following incubation with SCPPPQ1 (Fig. 3a). Fluorescence-activated cell sorting (FACS) analysis was also used to evaluate the effect of SCPPPQ1 on *P.* *gingivalis* over time. There was a significant reduction in the number of FACS events as soon as the protein was added (40% reduction at 0 min incubation) (Fig. 3c). During the first hour, this decrease gradually reached 75% and then remained constant over time (Fig. 3c). In contrast, as expected, the evolution of the bacterial population in the buffer without the protein progressively doubled during the same time lapse analysis (Fig. 3c), despite the presence of urea. Together, these two independent methods demonstrate a significant variation in bacterial behavior in presence of SCPPPQ1.

### Effect of SCPPPQ1 on formation of bacterial aggregates

**Figure 4:**
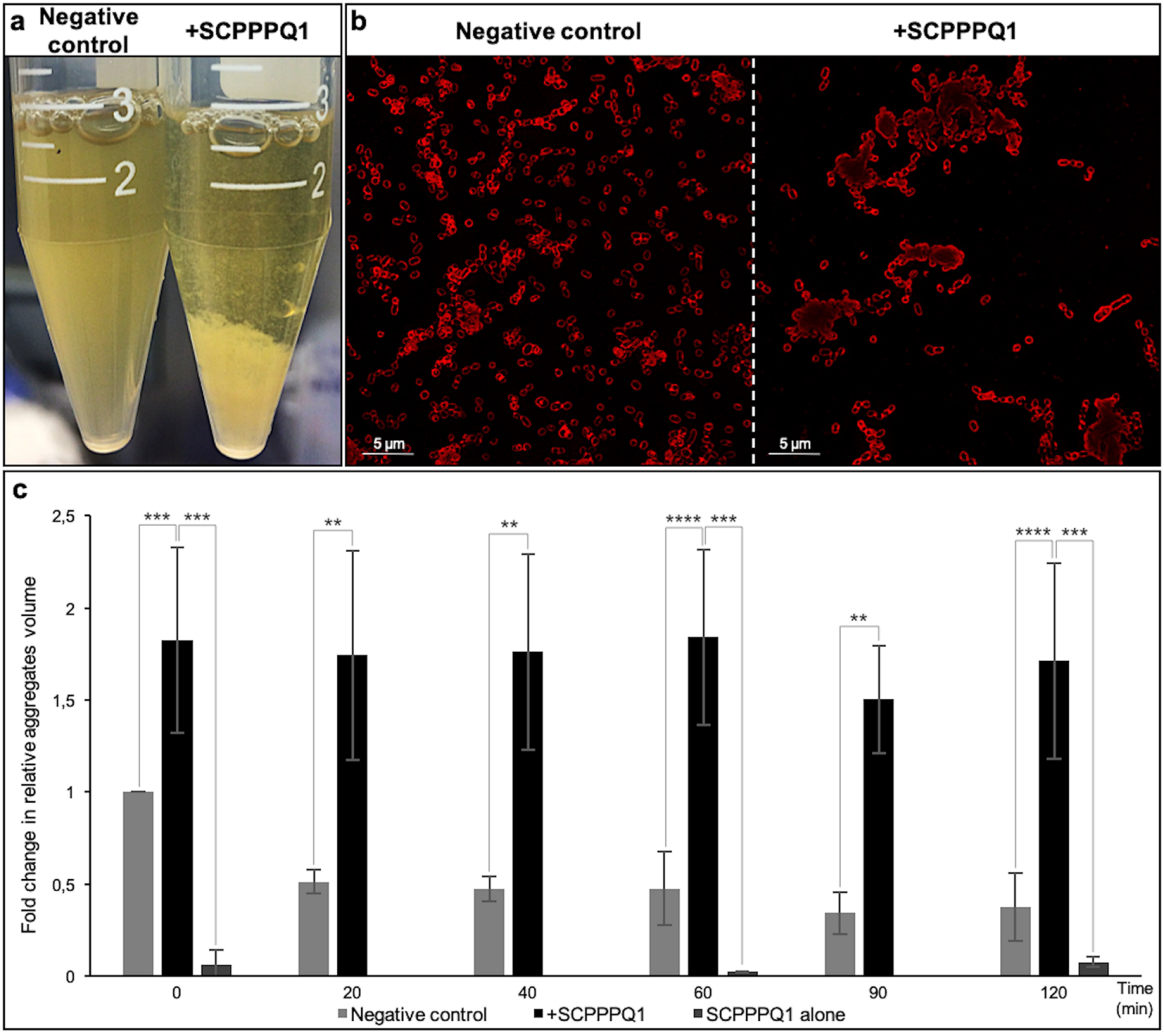
Effect of SCPPPQ1 on formation of *P.* *gingivalis* bacterial aggregates. (**a**) Visual inspection of bacterial cultures in test tubes from the negative control (left) and + SCPPPQ1 treated (right) samples at 20 min. A representative image from six different experiments is shown. (**b**) Super-resolution fluorescence images of bacteria (membrane stained in red with FM 4–64 dye) from the negative control (left) and + SCPPPQ1 treated (right) samples at 20 min. Images are representative from five different experiments. (**c**) FACS results of the fold change in relative aggregates volume over time normalized on the negative control at 0 min. Data are represented as mean ± standard error of mean (*n* = 4). Significance was determined by two-tailed Student’s *t* test analysis (**p < 0.01; ***p < 0.001; ****p < 0.0001).

To evaluate the propensity of SCPPPQ1 to induce formation of bacterial aggregates, the turbidity of the culture medium under the experimental and control conditions was compared. Addition of the protein to the bacterial suspension decreased its turbidity and increased precipitation and accumulation of material at the bottom of the tube as compared to adding buffer without protein (negative control) (Fig. 4a). Super-resolution fluorescence imaging revealed that precipitates, only formed in the presence of SCPPPQ1, consisted largely of aggregated bacteria (Fig. 4b). Furthermore, FACS analysis indicated that the relative volume of aggregates increased from almost two-fold at 0 min to four-fold at 120 min during incubation with SCPPPQ1 compared to the negative control (Fig. 4c). To eliminate a possible bias that could be introduced by the self-aggregation of the protein, SCPPPQ1 alone was similarly analysed by FACS at 0, 60 and 120 min. None or few fluorescent aggregates were detected (Fig. 4c). Details of the volume of these different aggregates are presented in Fig. S1a. In addition to there being more aggregates when bacteria are incubated with SCPPPQ1, aggregate volumes above 2.5 K on the FSC-H axis are significantly represented at 60 min (Fig. S1b). Similar results were obtained at the other time intervals (data not shown). Altogether, these results indicate that SCPPPQ1 promotes the formation of bacterial aggregates.

### Localisation of SCPPPQ1 on *P. gingivalis*

**Figure 5:**
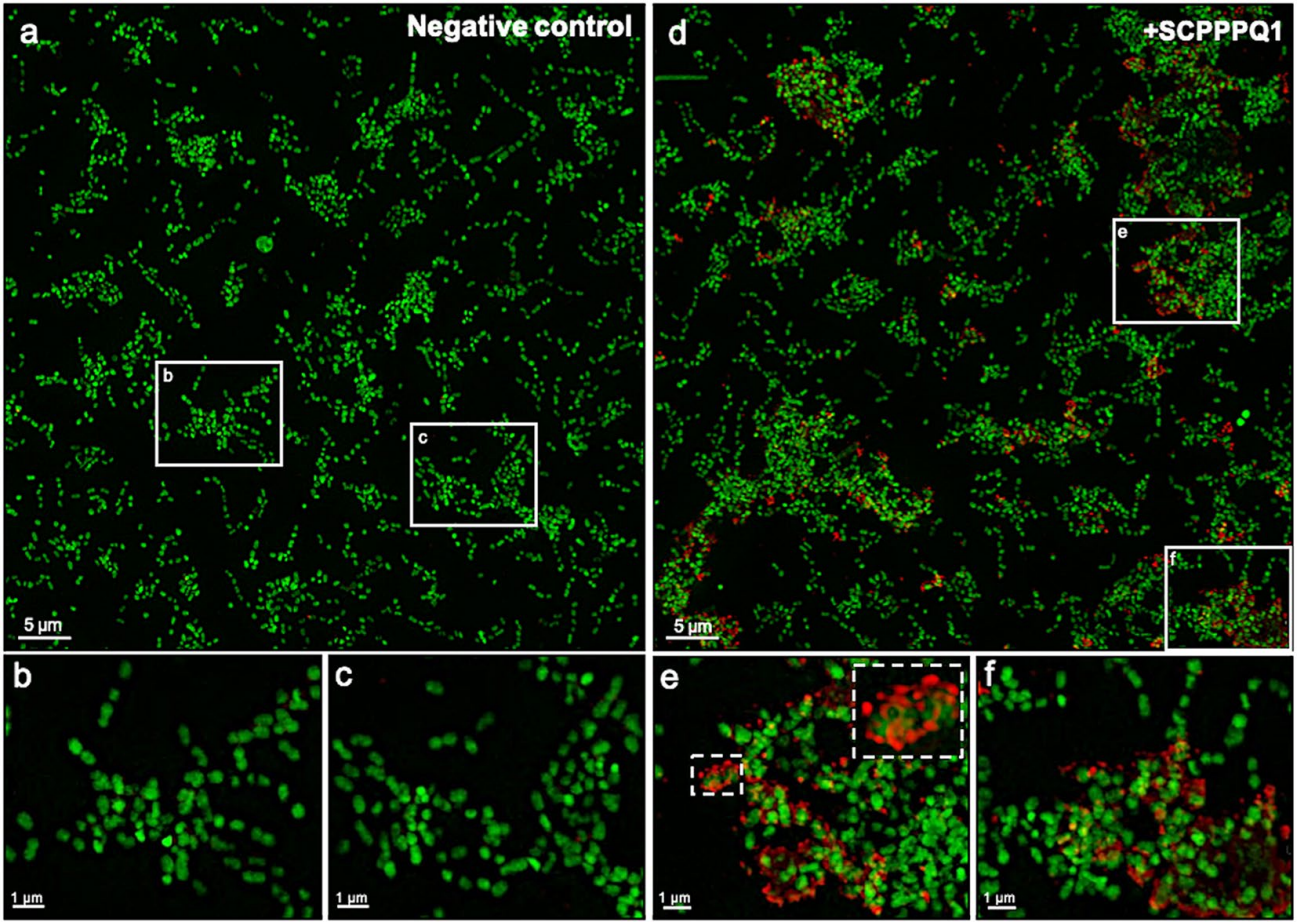
Localisation of SCPPPQ1 on *P.* *gingivalis* using super-resolution fluorescence imaging. (**a**–**c**) Images of bacteria incubated with the buffer only (negative control) at 20 min. (**b**,**c**) Enlargements of corresponding boxed areas in (**a**). (**d**–**f**) Images of bacteria incubated with + SCPPPQ1 at 20 min. (**e**, **f**) Enlargements of corresponding boxed areas in (**d**). (**e**) Upper dashed box is a magnification of the lower one. Bacterial nucleic acids were labeled with SYTO 9 (green fluorescence) and SCPPPQ1 with Alexa Fluor-546 secondary antibody (red fluorescence). These images are representative from four different experiments.

**Figure 6:**
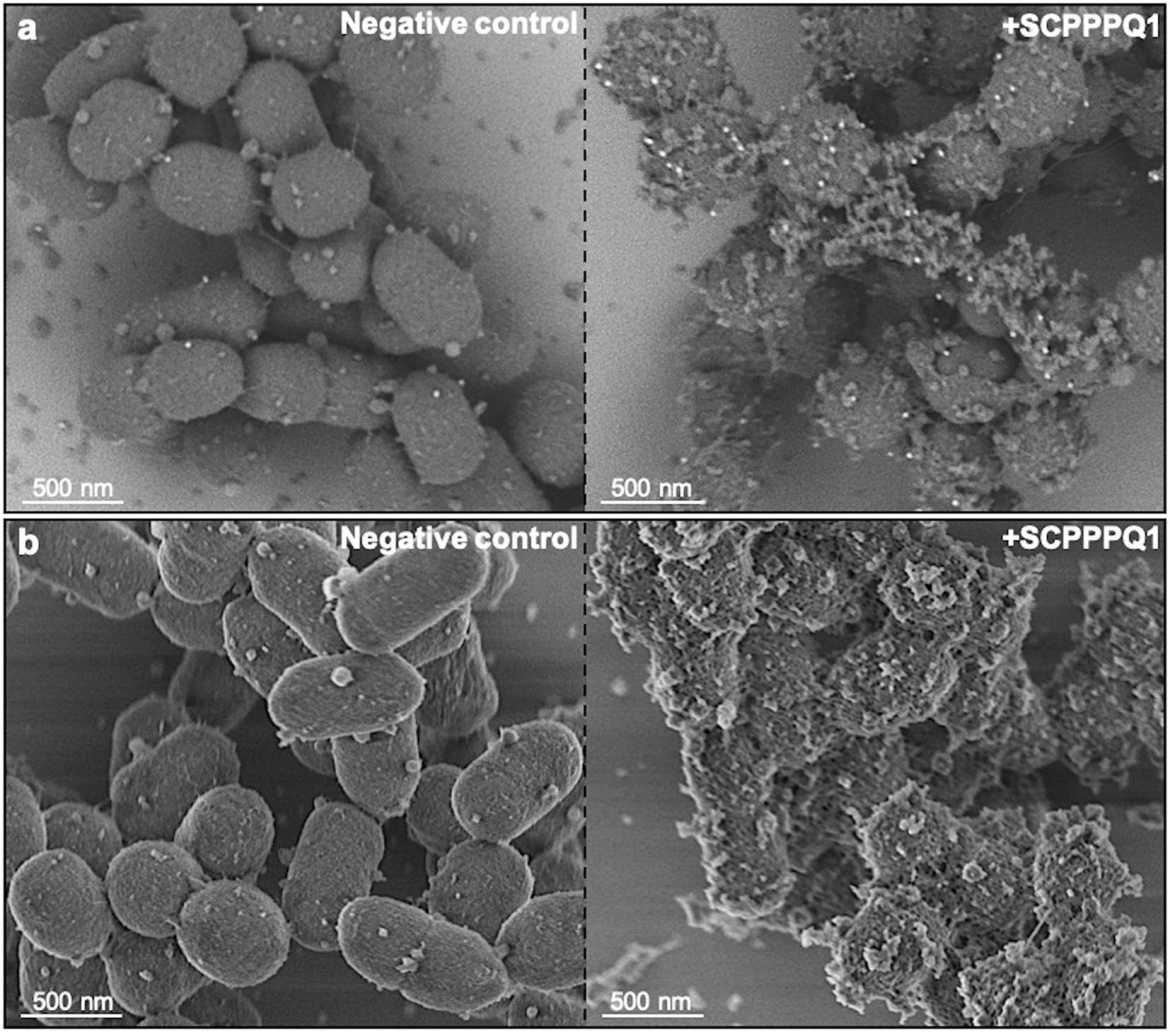
Localisation and effect of SCPPPQ1 on the surface of *P.* *gingivalis* using SEM. (**a**) SEM micrographs of colloidal gold immunolabeled preparation showing the presence of gold particles (white dot) on bacteria from samples treated with + SCPPPQ1 (right) and their paucity in the negative control (left) at 20 min. (**b**) SEM micrographs showing the normal appearance of the bacterial surface in the negative control (left) and its disruption of the bacterial surface in samples treated with + SCPPPQ1 (right) at 20 min. Micrographs are representative from four different experiments.

**Figure 7:**
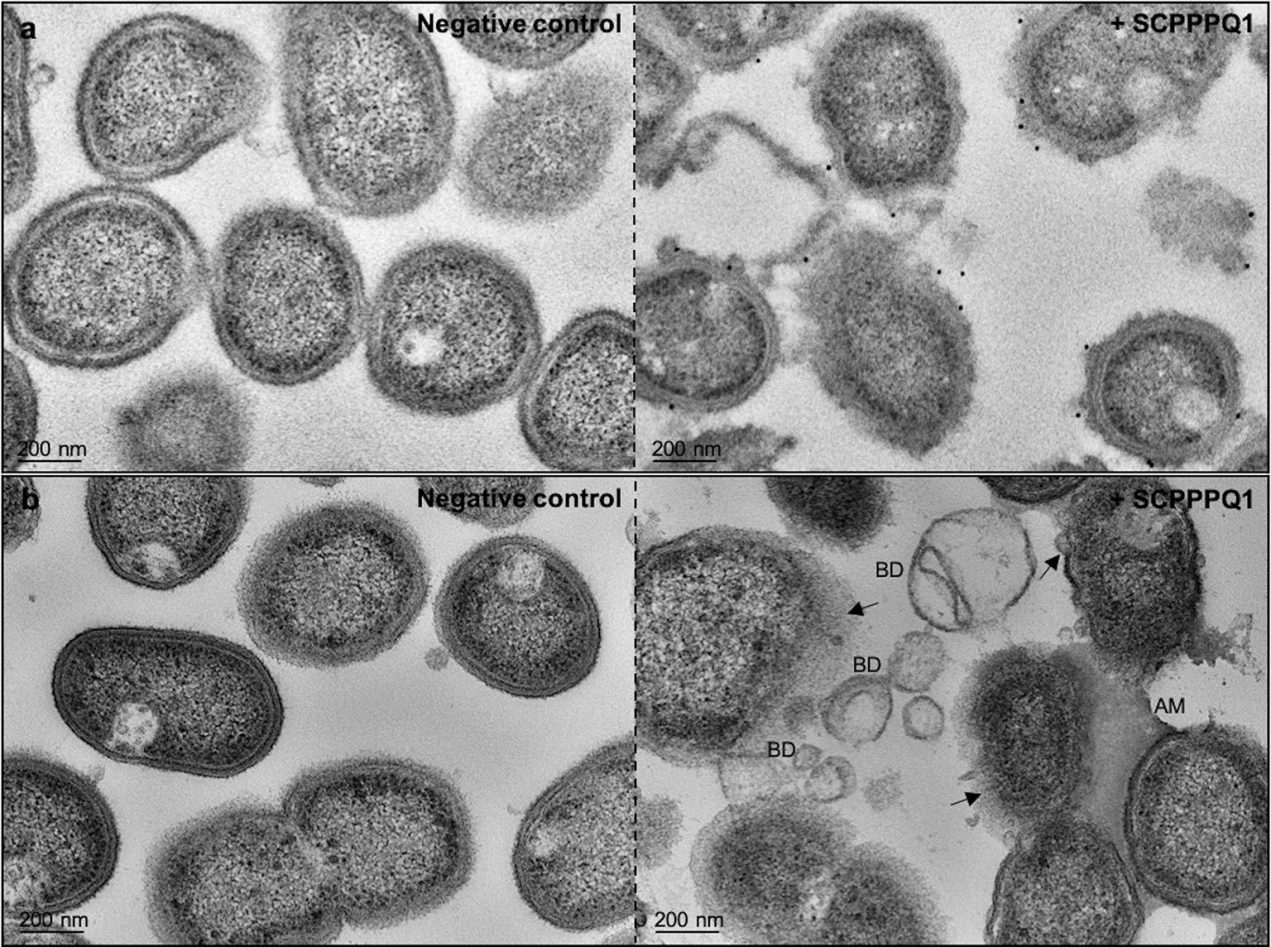
Localisation and effect of SCPPPQ1 on *P.* *gingivalis* bacteria using TEM imaging. (**a**) TEM micrographs of pre-embedded immunogold preparations of bacteria from negative control (left) and + SCPPPQ1 treated (right) samples at 20 min. No or very few gold particles (black dots) are present in the negative control while particles are associated with the bacterial surface in + SCPPPQ1 samples. (**b**) TEM micrographs of bacteria sections from the negative control (left) and + SCPPPQ1 treated (right) samples at 20 min. Following treatment with + SCPPPQ1, alteration of bacterial membrane (arrows), bacterial debris (BD) and aggregated material (AM) can be visualized. Micrographs are representative from three different experiments.

In order to evaluate binding of SCPPPQ1 to *P.* *gingivalis*, super-resolution fluorescence imaging was applied (Fig. 5). Protein was observed on the majority of bacterial aggregates and it accumulated focally causing a beaded fluorescence appearance around the bacteria (Fig. 5d–f). Labeling was absent and only bacteria were visible in negative control incubations (Fig. 5a–c). Colloidal gold immunolabelling for SCPPPQ1 was carried out and the presence of gold particles on the surface of bacteria was visualised by SEM (Fig. 6a). These preparations also allowed to observe the disruptive effect that the protein has on bacterial membrane integrity (Fig. 6b). Such membrane disruption was infrequently observed under control conditions (carrier buffer alone). Transmission electron microscope (TEM) analysis of pre-embedding colloidal gold labeled preparations confirmed the above SEM data (Fig. 7). In addition, TEM imaging revealed (*a*) the association of the protein with the bacteria surface/membrane (Fig. 7a), (*b*) the alteration of the membranes by the protein, which appeared disrupted and having an enlarged spacing (Fig. 7a,b), and (*c*) the presence of bacterial debris as well as fine granular extracellular material (Fig. 7b). Together, these results indicate a causal link between the interaction and surrounding of bacteria with SCPPPQ1 and their alteration.

### Effect of SCPPPQ1 on other microorganisms

**Figure 8:**
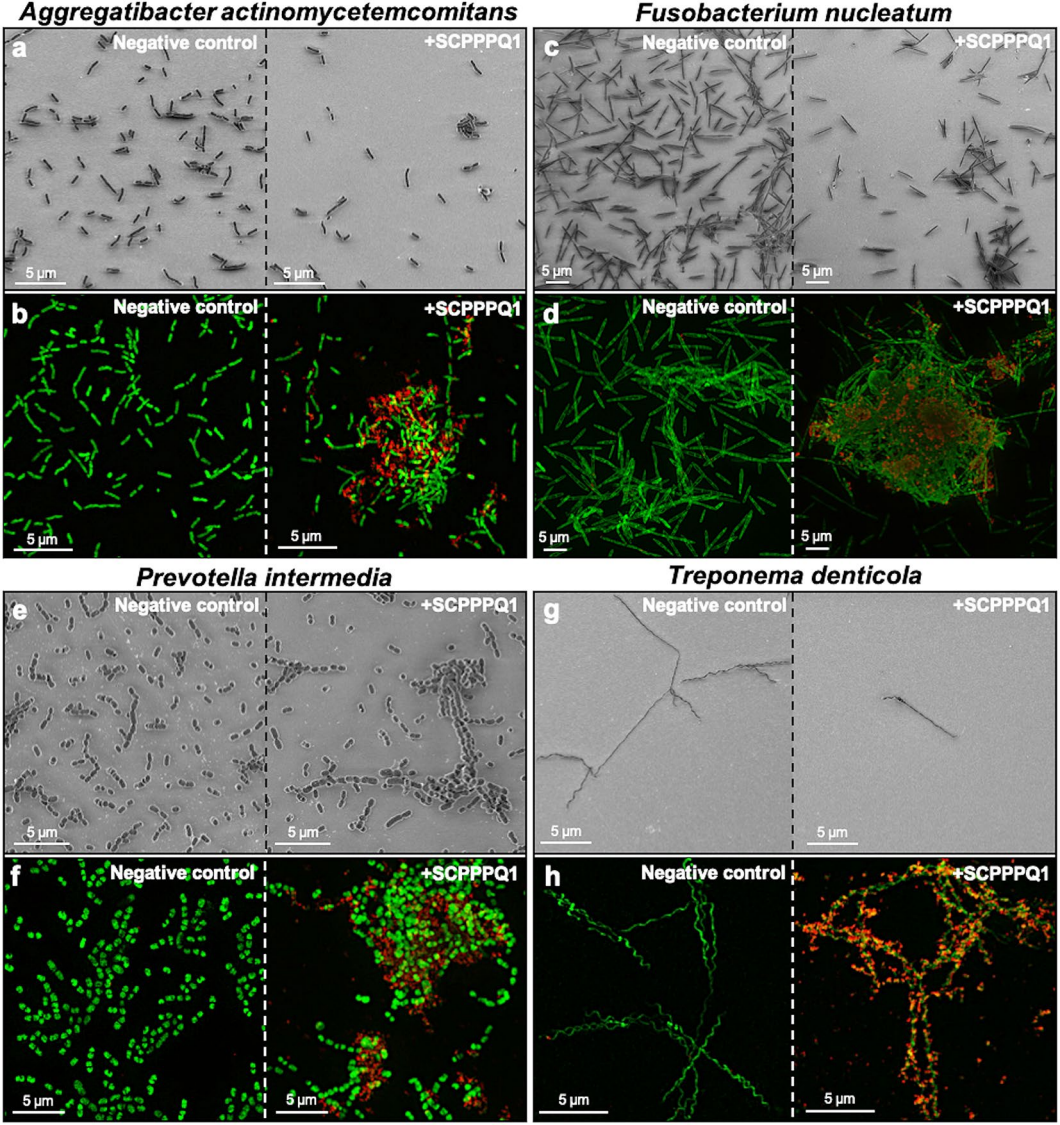
Effect and localisation of SCPPPQ1 on other oral bacteria. (**a,c,e**,**g**) SEM micrographs and (**b,d,f**,**h**) Super-resolution fluorescence images of (**a,b**) *A.* *actinomycetemcomitans,* (**c,d**) *F.* *nucleatum,* (**e,f**) *P.* *intermedia* and (**g,h**) *T.* *denticola* from the negative control (left) and + SCPPPQ1 treated (right) samples at 20 min. Bacterial nucleic acids were labeled with SYTO 9 (green fluorescence) and SCPPPQ1 with Alexa Fluor-546 secondary antibody (red fluorescence). Images are representative from three different experiments.

To determine the extent of antibacterial action of SCPPPQ1, its effect was also evaluated on other periodontopathogenic bacteria including *Aggregatibacter* *actinomycetemcomitans* *(A.* *actinomycetemcomitans)*, *Fusobacterium* *nucleatum* *(F.* *nucleatum),* *Prevotella* *intermedia* *(P.* *intermedia),* and *Treponema* *denticola* *(T.* *denticola)* (Fig. 8). As with *P.* *gingivalis*, SEM imaging showed fewer bacteria in preparations treated with SCPPPQ1 (Fig. 8a,c,e,g). Moreover, immunofluorescence revealed the presence of the protein on the bacterial cell surface (Fig. 8b,d,f,h), this being notably obvious with *T.* *denticola* (Fig. 8h). Both methods, also revealed an important presence of aggregates. The SCPPPQ1 protein therefore appears to have a similar effect on some other periodontal pathogens associated with the progression of PD.

### Antibacterial effect of peptides derived from SCPPPQ1

**Figure 9:**
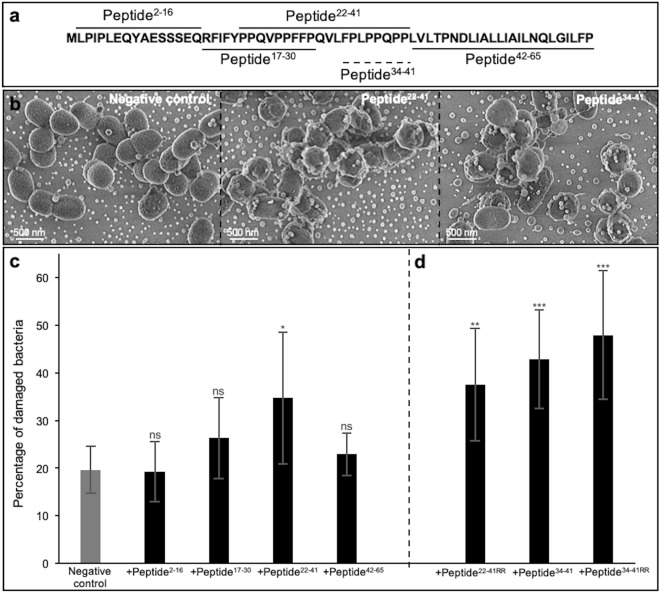
Effect of peptides on the membrane of *P.* *gingivalis*. (**a**) Amino acid sequence of full-length SCPPPQ1 and the corresponding five peptides generated. Peptide names are derived from the amino acid position in the full-length protein and are indicated in superscript. The dotted line represents the smallest peptide created. (**b**) SEM micrographs showing bacteria from the negative control,  + peptide^22–42^, and + peptide^34–41^ treated samples at 2 h. Micrographs are representative from five different experiments. (**c,d**) Quantification of the number bacteria with damaged membrane in percentage from the negative control and + peptides treated samples. Two peptides were synthesized in which two arginines were added on the C-terminal end; these are referred to as peptide^22-41RR^ and peptide^34-41RR^. Data are represented as mean ± standard error of mean (*n* = 5). Significance was determined by two-tailed Student’s *t* test analysis (ns: *p* > 0.05; *p < 0.05; **p < 0.01; ***p < 0.001).

To determine the active portion of SCPPPQ1 that lead to antibacterial effects, four peptides derived from its sequence were synthesised. These were designed to cover the complete protein sequence (Figs. 9a and S2a), with peptide^17–30^ and peptide^﻿22–41^ encompassing the disordered portion (residues 21 to 44) of the molecule, as predicted using GlobPlot2. Aggregate formation in presence of the peptides was visualised in the SEM. Although less obvious visually (data not shown) than with the full-length protein, quantification from SEM images indicated that peptide^2–16^ and peptide^17–30^ resulted in a significant presence of aggregates (Fig. S2b). SEM preparations also revealed bacterial membrane damage to *P. gingivalis* when incubated with the peptides and quantification showed that peptide^22–41^  affected a significant number of bacteria (Fig. 9b,c).

Cationic antimicrobial peptides (CAMPs) are well known to bind and penetrate bacterial cell membranes, leading to membrane disruption and ultimately cell death^15–17^. CAMPs are characterized by having less than 50 residues and a positive net charge^18^. In order to improve the efficiency of peptides in disrupting the bacterial membrane, two arginines (R) were added on the C-terminal end of selected peptides to increase their net positive charge^19^. We have thus modified peptide^22–41^, which has a charge of + 1 at neutral pH (http://PepCalc.com, from Innovagen AB), into peptide^22–41RR^ with a net positive charge of + 3 at neutral pH. This modification resulted in a more significant effect on the bacterial membrane integrity (Fig. 9d). Then to define the smallest effective size, peptide^22–41^ was reduced to 8 residues, FPLPPQPP (peptide^34–41^) (Fig. 9a). The latter has a net charge of + 1. Two arginines were added to its C-terminal to similarly obtain a final net charge of + 3 at neutral pH (peptide^34–41RR^). Peptide^34–41^ and peptide^34–41RR^ both appeared to have a significantly higher effect on membrane disruption (with peptide^34–41RR^^39^ showing a tendency for a higher efficiency), compared to peptides^22–41^ and ^22–41RR^ (Fig. 9c,d). Altogether the peptide findings indicate that the antibacterial capacity of SCPPPQ1 is defined by different portions of the protein that act in bacterial aggregation or membrane integrity.

## Discussion

It was recently reported that rat SCPPPQ1 has a marked effect on the membrane integrity of *P.* *gingivalis* that ultimately affects its growth^11^. A previous prediction for the human sequence of SCPPPQ1^20^ turned out to be somewhat different from the one decrypted using DNA extracted from human dental tissues and reported in GeneBank (#MK322956.1; https://www.ncbi.nlm.nih.gov/nuccore/MK322956.1). This sequence also has only 56% identity and 64% similarity with its rat homologue. Because of these differences, it was therefore important to validate and further explore the antibacterial potential and mode of action of human SCPPPQ1. The various assays carried out, showed that the *P.* *gingivalis* population rapidly and significantly decreases when incubated with human SCPPPQ1. Two mechanisms, aggregation of bacteria and membrane disruption, appear to be involved in this decrease. As we have previously shown, SCPPPQ1 tends to self-aggregate and intrinsically form molecular networks^9^, which we believe could promote bacterial ‘clumping’ and favor the interaction of SCPPPQ1 with the membrane of intact bacteria, ultimately affecting their integrity. In silico analyses, using two different prediction softwares, further indicated that like the rat protein, human SCPPPQ1 possesses potential antimicrobial peptide sequences.

Some AMPs are part of the innate immune system of both humans and animals and act as a first line of defence against a hostile environment^21^. They have broad-spectrum antimicrobial activities and possess modes of action that cannot be easily hijacked by the pathogen. The combination of small peptides with other AMPs or antibiotics for antibacterial applications^22–25^ is an emerging category of therapeutic agents. Such peptides have the advantages of being (a) easily synthesized and delivered, (b) more resistant, (c) less toxic and (d) more specific and selective^26^. Peptide^2–16^ and peptide^17–30^ were responsible for aggregation and, together, may determine the overall higher aggregating capacity demonstrated by the full-length protein. Regarding membrane disruption, our data points to peptide^34–41^ (FPLPPQPP) as the active portion of the protein. The resulting cell lysis can also help to reinforce bacterial clumping by releasing DNA extracellularly. Because of the “sticky” nature of free DNA, it can act as an agglutinin that promotes the formation of larger bacterial clumps^27^. Altogether, our data suggest a concerted antibacterial action caused by bacterial aggregation and membrane disruption sustained by different active portions of the molecule. In addition to *P.* *gingivalis*, we also have generated new evidence that other major bacteria implicated in PD are susceptible to human SCPPPQ1.

There are examples of AMPs with both antibacterial and antifungal properties such as Histatins produced by the parotid and submandibular glands^28^. In this context, we explored whether SCPPPQ1 has any effect on *Candida* *albicans* *(C.* *albicans),* a fungus present in the oral environment. Initial results show that samples exposed to SCPPPQ1 showed fewer fungi as compared with incubation with buffer only (Fig. S3). Hyphae were also more abundant in the control (Fig. S3). This is interesting since they have an important role in causing disease^29^. Thus, SCPPPQ1 could potentially also have both antibacterial and antifungal properties.

A challenge for antibiotics is to diffuse through biofilm and/or bacterial membranes, therefore biofilms bacteria show greater resistance to antibiotics^30^. In the case of the oral environment, *P.* *gingivalis* was found in up to 85% of dental plaque biofilms sampled from patients with PD^31–33^. Due to the natural presence of SCPPPQ1 in the mouth, its affinity to mineral tooth surface^20,34^ and its action against *P.* *gingivalis* and other dental plaque bacteria, this protein appears to be a prospective candidate to fight dental plaque biofilms. Its distribution in slow delivery systems and tooth paste, for instance, could be exploited to prevent bacterial attachment and accumulation on exposed tooth surfaces to limit biofilm formation. Since phosphoserine residues interact strongly with hydroxyapatite^35,36^, modifying our most effective peptides with a tooth-binding domain of diphosphoserine (Ser(p)-Ser(p)-) should probably increase their potential against dental biofilm^36^.

The findings we report here are novel and point to an interesting antimicrobial potential, however, our study has some limitations. While we have demonstrated that human SCPPPQ1 has an impact on both the population number and cell membrane integrity of *P.* *gingivalis*, we were unable to carry out a dose-dependent effect using a conventional MIC assay^37^ because we could not discriminate the effect of the protein from that of the Na_2_HPO_4_ buffer containing urea at final concentrations ≤ 1 M. However, a concentration-dependent response, albeit qualitative, was observed using a blood agar growth plate assay, which validated the 20 μM SCPPPQ1 concentration used in our various experiments.

The reason we have used a urea-containing buffer is that SCPPPQ1 does not dissolve readily. Urea is well known to have antibacterial properties^38^ and is often used for extracting proteins from the bacterial cell surface^39^, albeit at substantially higher molarity (e.g. 8 M vs ≤ 1 M) and with longer incubation period (e.g. 1 h vs 20 min) than we have used. Clearly this confounds interpretation of our results, but in all cases, they were contrasted to the urea-containing buffer control diluted in the same proportions. Also, the distinctive results obtained with the various peptides highlights an effect above and beyond any contribution by the buffer. Since it cannot be excluded that the observed antibacterial effect relates in part to urea, optimisation of the antibacterial activity of SCPPPQ1 and particularly therapeutic use will require a better adapted delivery vehicle. In fact, urea is used to isolate the crude bacterial envelope and bacterial outer membrane^39^ and it could even actually ‘prime’ the surface for SCPPPQ1 binding for a synergistic action. Irrespective of these limitations, the evidence derived from our multiple experimental approaches used shows that SCPPPQ1 has an effect on bacterial number and integrity.

In conclusion, our results brought evidence that human SCPPPQ1, a structural extracellular matrix protein, can affect various periodontal pathogens, more specifically *P.* *gingivalis*. Its mode of action against *P.* *gingivalis* has been demonstrated for the first time and implicates bacterial aggregation and membrane disruption. These lead to major membrane breaches that are bound to cause cell death. Using engineered peptides, we have further identified the active portions of SCPPPQ1 respectively implicated in these two processes. The integration of SCPPPQ1 or these peptides in oral hygiene products may offer novel therapeutic strategies for controlling the formation of biofilms associated with oral surfaces and dental implants^40^, limiting development of dental carries and PD, and ultimately for alleviating linked systemic complications. In a broader context, it could also offer an additional prospective to deal with the increasing challenge of bacterial resistance.

## Methods

### In silico analysis

CLUSTAL multiple sequence alignment tool Multiple Sequence Comparison by Log-Expectation, (MUSCLE version 3.8, https://www.ebi.ac.uk/Tools/msa/muscle/; EMBL-EBI, Hinxton, England)^41^ was used to compare the sequence of rat and human SCPPPQ1. The amino acid composition and the antimicrobial potential of SCPPPQ1 was determined using both AMP database APD3 (https://aps.unmc.edu; University of Nebraska, Omaha, NE, USA)^14^ and different prediction algorithms (iAmpPred (http://cabgrid.res.in:8080/amppred/index.html), and AmPEP and Deep-AmPEP30 (https://cbbio.online/AxPEP/))^12,13^. The Grand Average Hydropathicity Value (GRAVY) was measured using the software Sequence Manipulation Suite^42^ to obtain the hydropathic character of the SCPPPQ1 protein^43^. To predict the tridimensional conformation of proteins both software i-TASSER (https://zhanggroup.org/I-TASSER/) and Quark (https://zhanggroup.org/QUARK/) (University of Michigan, Ann Arbor, MI, USA) were used^44,45^. MacPyMOL version 2.0 (The PyMol Molecular Graphics System, Schrödinger, LLC) was used to analyse and align the predicted models of the 3D structures. The percentage of disordered and ordered domains were obtained with GlobPlot version 2.3 (EMBL, Heidelberg, Germany)^46^.

### Bacterial culture

*Porphyromonas* *gingivalis* (ATCC 33277, Manassas, VA, USA), *Aggregatibacter* *actinomycetemcomitans* (ATCC 29522), *Fusobacterium* *nucleatum* (ATCC 25586) and *Prevotella* *intermedia* (ATCC 25611) were grown anaerobically (80% N_2_, 10% CO_2_, 10% H_2_) in Todd-Hewitt broth (Thermo Fisher Scientific, Waltham, MA, USA) supplemented with 0.0001% vitamin K (Thermo Fisher Scientific) and 0.001% hemin (Thermo Fisher Scientific), for 48 h at 37 °C. Then the culture was diluted 1/10 (v/v) in the same medium and bacteria were grown anaerobically at 37 °C for 24 h. *Treponema* *denticola* (ATCC 35404) was grown anaerobically for 24 h in liquid medium consisting of 2 mg/mL of sodium bicarbonate (Thermo Fisher Scientific), 12.5 mg/mL of brain heart infusion (Thermo Fisher Scientific), 2 mg/mL of glucose (Thermo Fisher Scientific), 0.25 mg/mL of L-asparagine (Thermo Fisher Scientific), 1 mg/mL of L-cysteine (Thermo Fisher Scientific), 0.5 mg/mL of sodium thioglycolate (Thermo Fisher Scientific), 6 μg/mL of thiamine pyrophosphate (Sigma-Aldrich, Saint-Louis, MO, USA), 10 mg/mL of trypticase (Thermo Fisher Scientific) and 2.5 mg/mL of yeast extract (Thermo Fisher Scientific); 2% rabbit serum (Thermo Fisher Scientific); and 0.2% volatile fatty acids (Sigma-Aldrich) (0.5 ml each of DL-2-methylbutyric, isobutyric, isovaleric and valeric acids dissolved in 100 ml of 0.1 M KOH)^10^. The bacteria used in the various assays had an optical density at a wavelength of 660 nm (OD_660_) around 1.

### Protein and peptide production

All tests were performed using human SCPPPQ1 protein and derived peptides synthesised by LifeTein Inc (Somerset, NJ, USA) with amidation in the C-terminal for peptides. To be consistent with the rat protein produced in bacteria, we have included a methionine at the beginning of the protein sequence^11^. In some cases, two arginines were added at the C-terminal end of peptides to increase their total net charge. The bulk synthetic products were solubilized in 50 mM Na_2_HPO_4_ (Sigma-Aldrich) containing 6 M urea (Sigma-Aldrich) buffer at pH 7.

Several concentrations of protein ranging from 5 to 150 µM and peptides from 10 to 200 µM were screened to determine a minimal working concentration that yielded apparent effects. It was determined that a final concentration of 20 µM for the protein and of 150 µM for the peptides would be used for the analyses described below.

Several lots of SCPPPQ1 were commercially synthesized and used throughout the study period. For every lot, following solubilisation, the protein concentration was evaluated using a Biodrop (Montreal Biotech Inc., Kirkland, QC, Canada) and ranged between 114–259 µM. To achieve a final 20 µM concentration, the ‘stock’ proteins dilutions ranged between 5.7–12.9X. Consequently, the final urea concentration to which bacteria were exposed in the analyses ranged between 0.47–1.05 M, for both the protein incubations and matched negative control (buffer only).

### Growth plate assay

The test was carried out on 10% Sheep Blood agar petri plate (Thermo Fisher Scientific). As for the liquid cultures, the petri dishes were reduced beforehand for 48 h under anaerobic conditions. Bacteria in Todd-Hewitt broth were mixed with different concentrations of SCPPPQ1 (see above) or protein solubilisation buffer only, as negative control. For each condition, the exact same liquid volume was quickly spread on the surface of a blood agar petri plate. The plates were then incubated at 37 °C under anaerobic conditions for 4 days to allow bacterial growth.

### Scanning electron microscope (SEM) analysis

Bacteria were placed on polished titanium discs^47^ for 20 min at 37 °C. Then a solution of the same volume of either SCPPPQ1 (20 µM final concentration) or peptide (150 µM final concentration), or buffer only as negative control was added on top and incubated at 37 °C for 20 min for the protein and 2 h for the peptides. Samples were then fixed for 30 min at 4 °C using a solution of 4% paraformaldehyde (PFA) (Thermo Fisher Scientific) and 0.1% glutaraldehyde (Electron Microscopy Sciences, Hatfield, PA, USA) in 0.1 M phosphate buffer (PB), pH 7.3. After rinsing three times with 0.1 M PB, samples were incubated with a 1% aqueous osmium tetroxide (Electron Microscopy Sciences) for 1 h at room temperature (RT). They were then dehydrated through a graded ethanol series (30%, 50%, 70%, 80%, 90%, 95% and twice in 100%) and finally dried using a Critical Point Drier CPD300 (Leica Biosystems, Concord, ON, Canada). A Regulus 8220 field-emission SEM (Hitachi, High-Technologies, Tokyo, Japan) operated at 1 kV was used to visualize the effect of the protein or peptides on the bacteria. ImageJ (NIH, Bethesda, MD USA) was used to count bacteria on at least ten representative images per sample derived from five different experiments for a total of 50 images analysed. Each condition on a disc incubated with SCPPPQ1 had a corresponding negative control disc for comparison.

### Counting analysis methods

For Fig. 3b, data are represented in percentage of bacteria present on the surface of titanium discs for a total area of 7560 µm^2^. The total number of bacteria on the negative control disc was counted and considered as the 100% reference. Then, the bacteria of the disc incubated with the protein were also counted and compared to its negative control to determine the ratio between the control and the treated sample.

For Fig. S2b, to determine bacterial aggregation following incubation of bacteria with peptides, we used the same method of counting as described above to represent the percentage of bacteria in aggregate. Briefly, all bacteria (single and in aggregates) present on the disc were counted and then those present in aggregate were separately recounted. A ratio was then determined between the bacteria present in aggregate and the number of total bacteria on the disc for each condition.

For Fig. 9c,d, to determine the percentage of bacteria with damaged membrane after incubation of the bacteria with peptides, all the bacteria (intact and damaged) were counted on the disc, and only those with damaged membrane were separately recounted. For each condition, a ratio was calculated to determine the percentage of bacteria with damaged membrane comparing to the total number of bacteria on the disc.

### Flow cytometry analysis, Fluorescence-activated cell sorting (FACS)

Suspensions of bacteria were incubated with either a 20 µM protein solution or buffer only (negative control) for up to 2 h at 37 °C. At 0, 20, 40, 60, 90 and 120 min, 50 µL of the mixture were sampled and fixed with 1% glutaraldehyde. To evaluate the propensity of the protein alone to form aggregates the protein solution at a final concentration of 20 µM was also incubated for up to 2 h at 37 °C in the supplemented Todd-Hewitt broth and then fixed as for the bacteria at 0, 60 and 120 min. The suspensions were then stained using 5 µg/mL FM 4–64 dye final (Thermo Fisher Scientific) before analysis with the analyser FacsAria III SORP (Becton Dickinson Life Sciences, Franklin Lakes, NJ, USA). The data were analysed using the FlowJo program version 10.6.1 (Becton Dickinson Life Sciences). The data obtained in Figs. 3c and 4c are represented as fold change over the negative control (buffer only) at 0 min due to the sensitivity of the FACS and to the variations between experiments.

For the aggregate volume experiments (Fig. 4c and Fig. S1), the total of aggregates was subdivided into different volume categories in the same way for all the samples in order to arbitrarily and consistently characterize various size of aggregates (Fig. S1a). To determine variation in total aggregates volume, the percentage of number of events for each subdivided part were multiplied by the number designated by the FSC-H axis in order to obtain a value for each section (smaller aggregates 1 to bigger aggregates 8). Detailed comparative analysis of aggregate formation under all the tested conditions (protein alone, bacteria alone, and protein + bacteria) was only carried out, and data at 60 min was presented. The independent values were added to obtain the total aggregates volume. FACS results were obtained from four independent experiments.

### Fluorescence studies

Suspensions of bacteria were incubated with SCPPPQ1 (20 µM final concentration) or buffer only (negative control) at 37 °C during 20 min. Ten µL of the samples were mixed with 10 µL of ProLong Gold Antifade Mountant (Thermo Fisher Scientific) and 5 µg/ml final of FM 4–64 dye (Thermo Fisher Scientific). The mixtures were then placed on glass slides and mounted with a No 1.5 glass coverslips (Thermo Fisher Scientific). The samples were examined under super-resolution structured illumination microscopy (SIM) using an Elyra PS1 microscope (Carl Zeiss Microscopy, Oberkochen, Germany) under the following conditions: objective 63x/1.4 oil Plan Apo DIC III; working distance: WD 0.10 mm; oil immersion and an Andor iXon3 EMCCD DU-885 K camera (Oxford Instruments, Concord, MA, USA). Z-stack volumes, with a mean number of step of 65 and a step-size of 0.110 µm. SIM reconstruction was done on each z-slice before being processed for extended depth of focus using the Zen Black edition software version 14.0.18.201 (Carl Zeiss Microscopy).

### Immunofluorescence studies

A suspension of bacteria were incubated with SCPPPQ1 (20 µM final concentration) or the same volume of buffer only (negative control) on coverslips at 37 °C during 20 min. Samples were then fixed 30 min with 4% PFA and 0.1% glutaraldehyde. Coverslips were then rinsed three times with 0.1 M PB before blocking for 1 h with 5% milk in 0.1 M PB. Next, coverslips were incubated for 1 h in 0.5% milk in 0.1 M PB with 1:500 rabbit antibody anti-human SCPPPQ1 (GenScript, Piscataway, NJ, USA) at RT. They were rinsed three times in 0.1 M PB and incubated for 1 h with 1:1000 goat anti-rabbit Alexa Fluor-546 secondary antibody (Thermo Fisher Scientific) at RT. Coverslips were then rinsed three times in 0.1 M PB, incubated 15 min with 1:500 SYTO 9 green fluorescent nucleic acid stain (Thermo Fisher Scientific) and finally mounted with ProLong Gold Antifade Mountant for observation. The samples were examined under the same conditions as describe above. Briefly, Z-stack volumes were acquired using SIM on an Elyra PS1 microscope and reconstructed using the Zen Black edition software version 14.0.18.201 as described above in the *fluorescence* *studies* section.

### Immunogold studies

For immunogold studies analysed by SEM, bacteria were incubated with 20 µM (final concentration) of protein or the same volume of buffer only (negative control) on polished titanium discs at 37 °C for 20 min and then fixed 30 min with 4% PFA and 0.1% glutaraldehyde. After rinsing samples three times with 0.1 M PB, samples were blocked 1 h with 5% milk in 0.1 M PB. They were then incubated 1 h in 0.5% milk in 0.1 M PB with 1:500 rabbit anti-human SCPPPQ1 antibody at RT. They were rinsed three times in 0.1 M PB, followed by a second 1 h blocking step and incubated 30 min with 1:50 20 nm protein A-gold beads (UMC, Utrecht, Netherlands) at RT. After rinsing three times with 0.1 M PB, samples were again fixed 30 min with 4% PFA and 0.1% glutaraldehyde, rinsed, incubated in 1% aqueous osmium tetroxide for 1 h at RT, dehydrated at RT through an ethanol series (30%, 50%, 70%, 80%, 90%, 95% and twice in 100%) for 15 min at each step and finally dried using a CPD300. A Regulus 8220 operated at 1 kV to detect the protein on the bacteria.

For preembedding immunogold studies, bacteria were incubated with 20 µM (final concentration) of SCPPPQ1 or buffer only (negative control) at 37 °C for 20 min. Then, the same immunolabeling protocol as described above for titanium discs was applied, except that between each step the sample was centrifuged for 4 min at 7,500 rcf. After the 100% ethanol step, the bacteria were processed for embedding in LR-white (Electron Microscopy Sciences) as follows: 3 parts of pure ethanol and 1 of resin (6 h, 4 °C), 2 parts pure ethanol and 2 parts resin (overnight, 4 °C), 1 part pure ethanol and 3 parts resin (12 h, 4 °C), pure resin (overnight, 4 °C), 100% resin (6 h, 4 °C), and 100% resin (72 h, 65 °C for curing). Ultrathin sections of 80–110 nm in thickness were cut with a diamond knife and transferred onto Formvar-coated (polyvinyl formate) 200-mesh nickel grids (Electron Microscopy Sciences) for imaging. Grids were stained with uranyl acetate and then examined with a Tecnai 12 (FEI, Eindhoven, Netherlands) transmission electron microscope (TEM) operated at 80 kV.

### Statistical tests

For SEM and FACS, mean values and standard error of mean were calculated from at least three independent experiments. *p* values were obtained by a two-tailed Student’s *t* test analysis of each condition from data in excel spreadsheet. Statistical significance was defined as *p* < 0.05 (*), *p* < 0.01 (**) and *p* < 0.001 (***).

## Supplementary Information

Supplementary Information 1.

Supplementary Information 2.

Supplementary Information 3.

Supplementary Information 4.
