# Supplementary material for: Effect of human secretory calcium-binding phosphoprotein proline-glutamine rich 1 protein on Porphyromonasgingivalis and identification of its active portions
Source: Sci Rep. 2021 Dec 9;11:23724. doi: 10.1038/s41598-021-02661-w (PMC8660882; doi:10.1038/s41598-021-02661-w)
Supplement: Supplementary file 1 — Supplementary Information 1. [file 41598_2021_2661_MOESM1_ESM.docx]

**Effect of human secretory calcium-binding phosphoprotein proline-glutamine rich 1 protein on** ***Porphyromonas gingivalis* and identification of its active portions**

Charline Mary^1,2^, Aurélien Fouillen^1,2^, Pierre Moffatt^3,4^, Dainelys Guadarrama Bello^1^, Rima M. Wazen^1^, Daniel Grenier^5^ and Antonio Nanci^1,2,6*^

^1^ Laboratory for the Study of Calcified Tissues and Biomaterials, Faculty of dental medicine, Université de Montréal, Montréal, Québec, Canada, H3T 1J4

^2^ Department of Biochemistry and Molecular Medicine, Faculty of Medicine, Université de Montréal, Montréal, Québec, Canada, H3T 1J4

^3^ Department of Human Genetics, McGill University, Montreal, Québec, Canada, H3A 0G4

^4^ Shriners Hospitals for Children - Canada, Montreal, Québec, Canada, H4A 0A9

^5^ Oral Ecology Research Group, Faculty of dental medicine, Université Laval, Québec (Québec), Canada G1V 0A6

^6^ Lead contact

* Corresponding author: Antonio Nanci

Email: [antonio.nanci@umontreal.ca](mailto:antonio.nanci@umontreal.ca)

**Keywords**

SCPPPQ1, Antibacterial peptides, Periodontal pathogens, Electron microscopy, Fluorescence microscopy

**Supplemental Experimental Details and Results**

**Determination of minimum inhibitory concentration (MIC)**

The minimum inhibitory concentration (MIC) of SCPPPQ1 was determined using a broth microdilution assay as routinely performed in our laboratory ^1^. Briefly, a 24-h culture of *P. gingivalis* ATCC 33277 was diluted in fresh Todd-Hewitt broth (THB; Becton Dickinson and Company, Sparks, MD, USA) supplemented with 0.001 % (w/v) hemin and 0.0001 % (w/v) vitamin K (THB-HK) to obtain an optical density at 660 nm (OD_660_) of 0.1, which corresponds to a concentration of 1 x 10^7^ colony forming units (CFU)/ml. Equal volumes (100 μl) of the bacterial suspension and two-fold serial dilutions of SCPPPQ1 (2.5 to 40 μM) in fresh medium were mixed in the wells of a 96-well, flat-bottom, microplate (Sarstedt, Newton, NC, USA). Control assays using the carrier buffer alone (no SCPPPQ1) or chlorhexidine (positive antibacterial molecule) were also performed. The microplate was incubated for 24 h in an anaerobic chamber (80 % N_2_, 10 % CO_2_, 10 % H_2_) at 37 °C prior to assessing bacterial growth by recording the OD_660_ using a Synergy 2 microplate reader (BioTek Instruments, Winooski, VT, USA). The MIC value was defined as the lowest concentration of the SCPPPQ1 that completely prevented bacterial growth. The MIC assay was performed in triplicate in two independent experiments.

**Results**

The MIC value of SCPPPQ1 against *P. gingivalis* was 40 µM, which corresponds to the highest concentration tested. However, at this concentration, the control assay using only the carrier buffer also completely inhibited bacterial growth. The MIC of the positive inhibitory compound (chlorhexidine) was 10 µM (5 µg/ml).

**Conclusion**

The carrier buffer exerts an antibacterial effect on *P. gingivalis* and therefore may complicate evaluation of the antibacterial property of SCPPPQ1 *per se*.

**Killing assay of planktonic bacterial cells**

The killing of planktonic cells of *P. gingivalis* ATCC 33277 by SCPPPQ1 was assessed using a previously described protocol used in our laboratory with slight modifications ^2^. Equal volumes of bacteria (final concentration of 10^5^ colony forming units (CFU)/ml) and SCPPPQ1 (80 µM in the carrier buffer) were mixed. In control assays, bacteria were incubated in the presence of the carrier buffer, 50 mM phosphate-buffered saline (negative control), or chlorhexidine (20 µM; positive antimicrobial molecule). Following a 20-min exposure at 20 °C in the absence of oxygen, ten-fold serial dilutions (10^-1^ to 10^-5^) were prepared in PBS. The presence of viable bacteria in each dilution was determined by transferring the dilutions into bacterial culture medium (THB-HK). To determine the log_10_ reduction, bacterial growth was visualized after two days of incubation (37 °C) in an anaerobic chamber (80 % N_2_, 10 % CO_2_, 10 % H_2_). The killing assay was performed in triplicate in two independent experiments.

**Results**

**Conditions log_10_ reduction**

*P. gingivalis* + PBS 0

*P. gingivalis* + SCPPPQ1 (80 µM) 2

*P. gingivalis* + Carrier buffer 2

*P. gingivalis* + Chlorhexidine (20 µM) 5

**Conclusions**

The carrier buffer caused a partial killing of *P. gingivalis* (20-min exposure) and therefore hinders evaluation of the antibacterial property of SCPPPQ1 *per se*.

***Candida albicans* analysis**

*C. albicans* (ATCC 36802; American Type Culture Collection) was cultured for 48 h in a media containing 17 g of Trypticase-Peptone (BD Biosciences), 3 g of yeast extract (BD Biosciences), 5 g of NaCl (Thermo Fisher Scientific), 2.5 g of disodium phosphate (Thermo Fisher Scientific), and supplemented with 1.5% (w/v) glucose (Thermo Fisher Scientific). The *C. albicans*culture was then diluted with culture media to a final concentration of ~105 cells/mL with the presence of human SCPPPQ1 at a final concentration of 20 µM or in the presence of the solubilizing buffer only (negative control). The 20 µM concentration was chosen because it matched the concentration of Farnesol used to generate a stress-response in *C. albicans* ^3^. A volume of 1,500 µl of each suspension were placed in each well of a 12-well plate containing titanium discs and incubated for 24 h. Samples were then fixed with a solution of 2.5% glutaraldehyde in 0.1 M PB, before processing for SEM visualization. This experiment was performed two times.

**Supplemental Figures**

**Figure S1. Size distribution of aggregate volumes of *Porphyromonas gingivalis*.**

**(a)** Typical flow cytometric dot plot pattern analysis showing the gating strategy used to identify FM 4-64 stained bacteria alone and aggregates.

**(b)** Representative results of the different aggregates volumes in percentage from fluorescence-activated cell sorting (FACS) gated data after incubation of *P. gingivalis* with either the buffer only (negative control) or +SCPPPQ1, and of the protein without *P. gingivalis* (SCPPPQ1 alone). Data are represented as mean ± standard error of mean (*n* = 4). Significance was determined by two-tailed Student’s *t* test analysis (ns: *p*> 0.05; *: *p*< 0.05; **: *p*< 0.01; ***: *p*< 0.001; ****: *p*< 0.0001).

**Figure S2. Effect of SCPPPQ1 on *Candida albicans*.**

Scanning electron microscope (SEM) images of *Candida albicans* from the negative control (left) and +SCPPPQ1 treated (right) samples at 24 hours. Note the presence of hyphae only in the negative control (arrows). Micrographs are representative from three different experiments.

**Figure S3. Effect of peptides derived from SCPPPQ1 on the formation of *Porphyromonas gingivalis* bacterial aggregates.**

**(a)** Amino acid sequence of full-length SCPPPQ1 and the corresponding four peptides generated. Peptide names are derived from the amino acid position in the full-length protein and are indicated in superscript.

**(b)** Percentage of bacterial aggregates in the negative control and +peptide treated samples quantified from SEM images at 2 hours. Data are represented as mean ± standard error of mean (*n* = 5). Significance was determined by two-tailed Student’s *t* test analysis (ns: *p*> 0.05; *: *p*< 0.05; **: *p*< 0.01).

References :

1 Ben Lagha, A., LeBel, G. & Grenier, D. Tart cherry (Prunus cerasus L.) fractions inhibit biofilm formation and adherence properties of oral pathogens and enhance oral epithelial barrier function. *Phytother Res* **34**, 886-895, doi:10.1002/ptr.6574 (2020).

2 LeBel, G., Vaillancourt, K., Morin, M. P. & Grenier, D. Antimicrobial Activity, Biocompatibility and Anti-inflammatory Properties of Cetylpyridinium Chloride-based Mouthwash Containing Sodium Fluoride and Xylitol: An *In Vitro* Study. *Oral Health Prev Dent* **18**, 1069-1076, doi:10.3290/j.ohpd.b871071 (2020).

3 Langford, M. L., Hasim, S., Nickerson, K. W. & Atkin, A. L. Activity and toxicity of farnesol towards *Candida albicans* are dependent on growth conditions. *Antimicrob Agents Chemother* **54**, 940-942, doi:10.1128/AAC.01214-09 (2010).
