# Supplementary figures and images for: Effect of human secretory calcium-binding phosphoprotein proline-glutamine rich 1 protein on Porphyromonasgingivalis and identification of its active portions
Source: Sci Rep. 2021 Dec 9;11:23724. doi: 10.1038/s41598-021-02661-w (PMC8660882; doi:10.1038/s41598-021-02661-w)

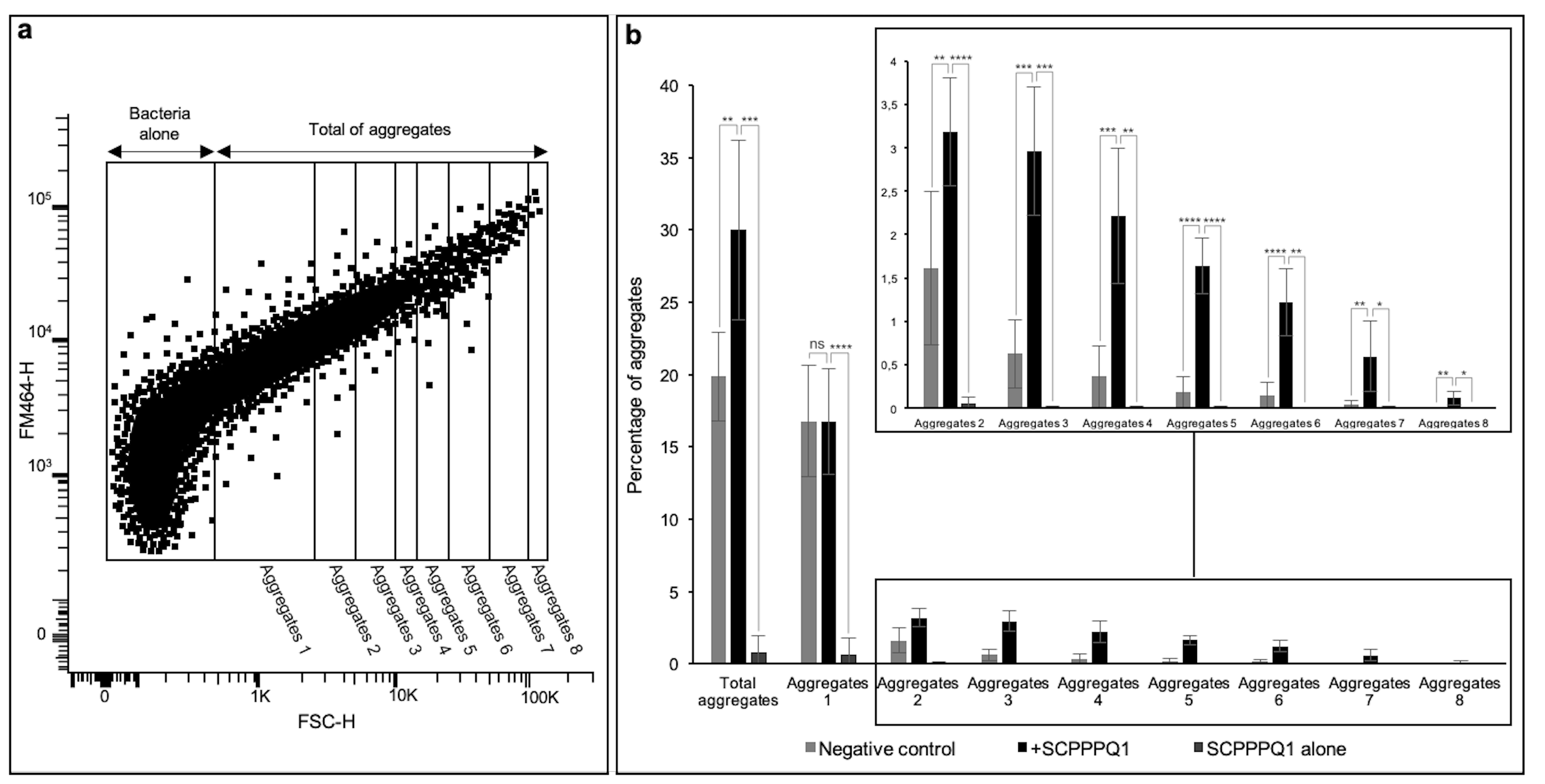

Supplement: Supplementary file 2 — Supplementary Information 2. [file 41598_2021_2661_MOESM2_ESM.tiff]

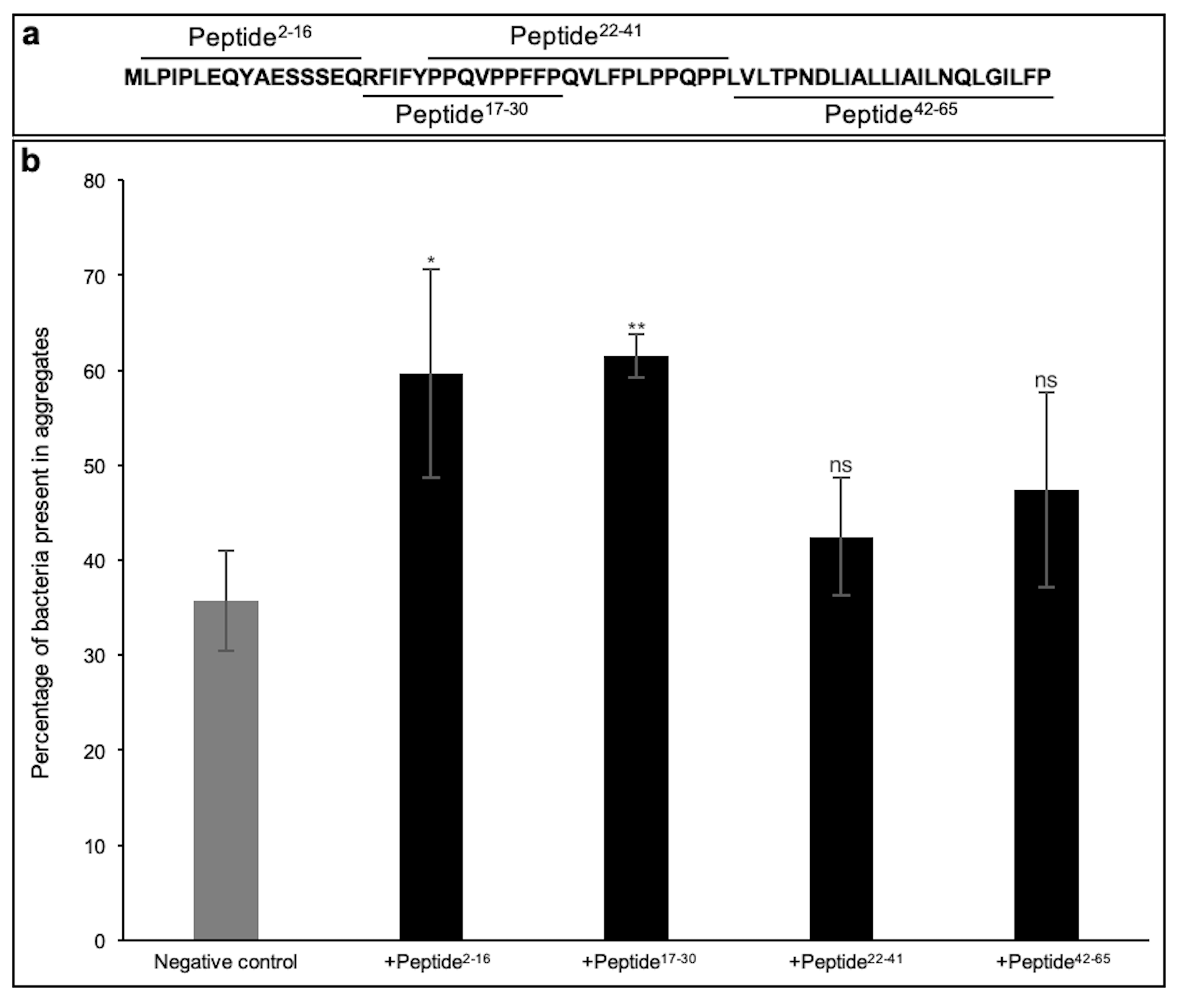

Supplement: Supplementary file 3 — Supplementary Information 3. [file 41598_2021_2661_MOESM3_ESM.tiff]

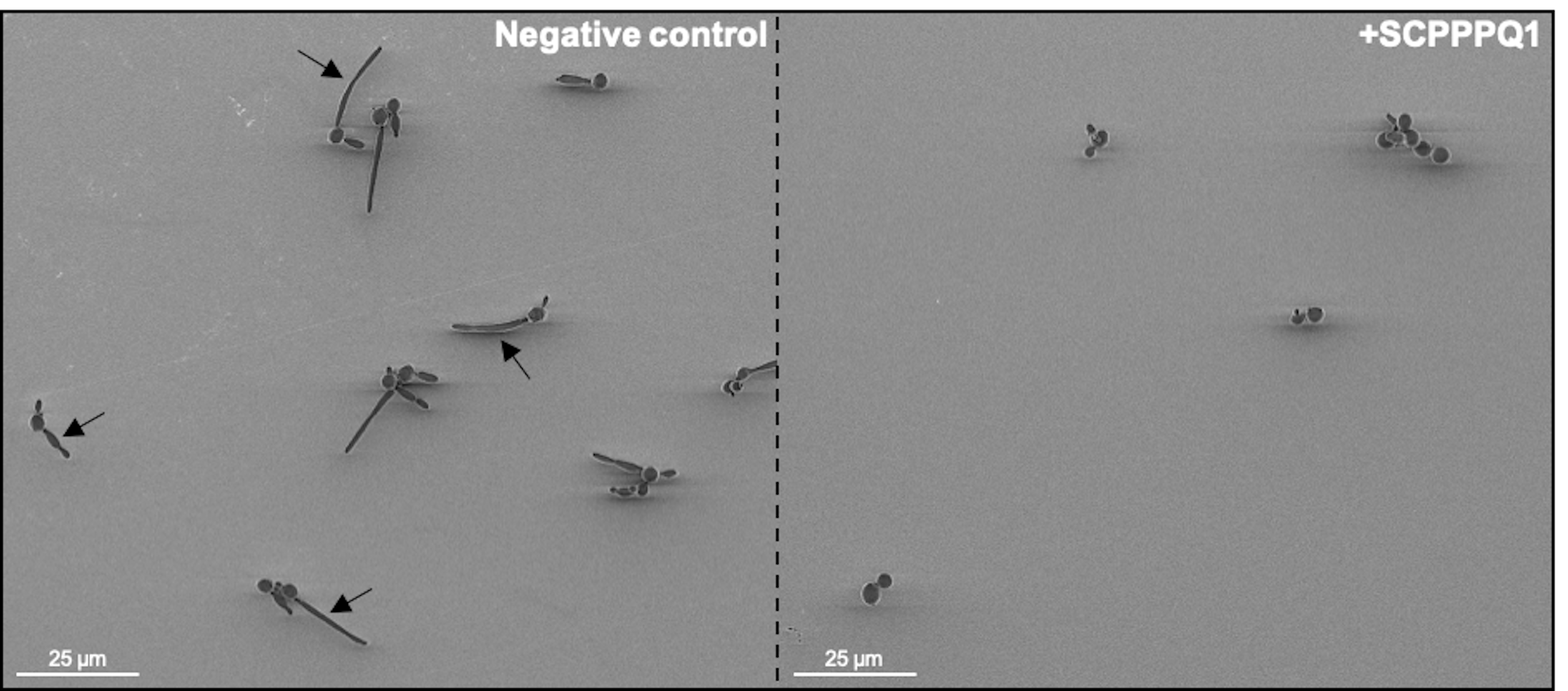

Supplement: Supplementary file 4 — Supplementary Information 4. [file 41598_2021_2661_MOESM4_ESM.tiff]
